# Supplementary material for: Steps against the burden of Parkinson’s disease (StepuP): Protocol of a randomized controlled trial elucidating the biomechanical and neurophysiological mechanisms of a speed dependent treadmill training intervention
Source: PLoS One. 2026 Jun 25;21(6):e0348957. doi: 10.1371/journal.pone.0348957 (PMC13298929; doi:10.1371/journal.pone.0348957)
Supplement: S1 File — (PDF) [file pone.0348957.s001.pdf]

Supplementary Table 1. Inclusion-exclusion questionnaire for healthy participants in Amsterdam

If an answer was "yes" on any of the questions printed in "bold" participants were excluded from participation.

Topic	Question	No	Yes	Additional comment
Walking ability	Do you use a walking aid (if so, what and how often)?			
	**Is it difficult for you to walk for 7 minutes at a time without resting?**			
	**Is it difficult for you to walk for 17 minutes in one session without an aid but with breaks in between?**			
	Do you experience pain when walking?			
	Do you experience more pain when walking for longer periods?			
Risk of falling	Have you fallen in the past year? (If yes, how often)?			
	Have you fallen more than twice in the past year?			
Neurological and psychiatric disorders	**Do you have Parkinson's disease?**			
	**Do you ever experience tingling or numbness in your hands, feet, or legs (for example, neuropathy due to diabetes)?**			
	**Do you have any neurological complaints for which you have visited a neurologist in the past year (if so, what were they for)?**			
	**Do you have a psychiatric condition?**			
Medication	**Have you used sleeping pills or tranquilizers in the past week? (benzodiazepines such as oxazepam, temazepam, diazepam, zoplidem, etc.)**			
	**Have you used antidepressants in the past week?**			
	**Have you used beta-blockers in the past week?**			
	**Have you used antiepileptic drugs (such as carbamazepine, clonazepam, levetiracetam, sodium valproate, etc.) in the past week?**			
Joint disorders	**Do you suffer from osteoporosis (bone loss) or osteoarthritis (joint wear and tear)?**			
	**Do you suffer from rheumatoid arthritis (joint inflammation)?**			
	**Do you have an artificial joint (hip or knee prosthesis)?**			
Lower extremity injury	**Did you break your leg last year?**			
	**Do you suffer from rheumatoid arthritis (joint inflammation)?**			
	**Did you tear your knee or ankle ligaments last year?**			
Vestibular	**Do you often feel dizzy?**			

disorders<br>Cardiovascular problems	Have you ever had a cardiac arrest and/or bypass surgery?			
	Have you ever had chest pain?			
	**Have you ever had stroke?**			
	Have you ever had a pulmonary embolism?			
	Do you have high blood pressure (systolic >140, diastolic >90) and/or are you taking medication for high blood pressure?			
	Do you have high cholesterol (>5.2) and/or are you taking medication for high cholesterol?			
	Have you ever fainted in the past 6 months?			
	Do you have any other heart problems<br>(e.g., palpitations, heart murmur, shortness of breath) for which you have visited a cardiologist in the past year (if yes, for what)?			
Vision and hearing	Do you have trouble reading the newspaper<br>(possibly with glasses or a magnifying glass)?			
	Do you have trouble recognizing someone's face from a distance of 4 meters (possibly with glasses)?			
	Do you have trouble hearing my questions clearly?			
